# Clinico-Pathological and Radiological Spectrum of Mediastinal Masses in a Tertiary Care Center: A Cross-Sectional Study

**DOI:** 10.7759/cureus.37922

**Published:** 2023-04-21

**Authors:** Shahana B, Irfan Ismail Ayub, Dhanasekar T, Sandhya Sundaram

**Affiliations:** 1 Pulmonology and Critical Care, Sri Ramachandra Institute of Higher Education and Research, Chennai, IND; 2 Pathology, Sri Ramachandra Institute of Higher Education and Research, Chennai, IND

**Keywords:** cross-sectional study, mediastinal compartments, pathological diagnosis, mediastinal tumour, mediastinal mass

## Abstract

Introduction

The phrase “mediastinal mass” refers to a mass within the mediastinum. About 50% of all mediastinal masses, including teratoma, thymoma, lymphoma, and thyroid illness, are anterior mediastinal tumors. Data on the mediastinal mass in India are relatively sparse, especially in this region, compared to those from other countries. Mediastinal masses are very infrequent lesions that might occasionally present a diagnostic and therapeutic challenge to the doctor. The current study describes the socio-demographic characteristics, symptoms, diagnosis, and location of mediastinal mass among the study participants.

Methodology

We carried out a retrospective, cross-sectional study in a tertiary care center in Chennai for three years. We included patients with an age above 16 years who visited the tertiary care center in Chennai during the study period. We included all patients with a mediastinal mass diagnosed by CT scan, with or without signs and symptoms of mediastinal compression. Patients under the age of 16 and those with insufficient data were both excluded from the study. As per the universal sampling technique, we included all the patients who met the eligibility criteria during the study period (three years) as study subjects. By using the hospital records, we collected all data about the patients like socio-demographic data, presenting complaints, past history, x-ray findings, and co-morbidities. Similarly, we recorded blood parameters, pleural fluid parameters, and histopathological reports from the laboratory register.

Results

The mean age of the study participants was 41.11 years, with a high proportion of patients belonging to the age group of 21 to 30 years. Over 70% of the study participants were male. Only about 54.5% of the study participants had symptoms because of a mediastinal mass. The most common local symptom felt by the patients was dyspnea, followed by a dry cough. Weight loss was the most common symptom for the patients. Most study participants (47.7%) had seen a doctor within one month of the onset of symptoms. About 4.5% of the patients had pleural effusion, as diagnosed by x-ray. Most of the study participants had a mass in the anterior mediastinum, followed by the posterior mediastinum. Most of the participants (15.9%) had non-caseating granulomatous inflammation suggestive of sarcoidosis.

Conclusion

The most common tumor found in our study was lymphoma, which was followed by non-caseating granulomatous disease and thymoma. Anterior compartments are most commonly involved. We observed the most common presentation in the third decade of life with a male to female ratio of 2:1, with dyspnea being the most common symptom, followed by a dry cough. Our study found 4.5% of the patients had pleural effusion as a complication.

## Introduction

Primary tumors, metastatic tumors, cysts, and acute and chronic infections are a few examples of mediastinum diseases. Only 3% of chest cancers are primary mediastinal tumors, which are uncommon [1]. The phrase “mediastinal mass” refers to a mass within the mediastinum. There is a wide variety of histology and radiological mediastinal mass lesions. The thoracic inlet superiorly, the pleural cavities laterally, and the diaphragm inferiorly define the mediastinum.

Many anatomists further divide it into posterior, anterior, and middle compartments. About 50% of all mediastinal masses, including teratoma, thymoma, lymphoma, and thyroid illness, are anterior mediastinal tumors [2,3]. Thymoma, lymphoma, and germ cell cancers like seminoma, teratoma, and yolk sac tumors all can cause a mass in the anterior mediastinum. The likelihood of malignant masses in this location is higher than in other compartments [4,5]. Thymomas, primary anterior mediastinal tumors that are more prevalent in adults (20%), seldom affect children [6].

Growth in the mediastinal space will cause a life-threatening emergency because of the space’s constriction and inability to expand. When a mass in the mediastinum grows large enough and presses on nearby organs, most mediastinal masses are slow-growing, and patients frequently seek medical attention. The complications of mediastinal mass are compression of the spinal cord, spread to nearby structures like the heart and great vessels (vena cava and aorta). Cough, chest pain, fevers/chills, and dyspnea are the most prevalent symptoms at presentation. We can divide most symptoms into two categories, such as localizing and systemic symptoms. Tumor invasion caused localizing symptoms. Respiratory compromise, paralysis of the limbs, dysphagia, vocal cords, and diaphragm dysfunction, superior vena cava syndrome, and Horner syndrome are common localizing symptoms.

Data on the mediastinal mass in India are relatively sparse, especially in this region, compared to those from other countries. Mediastinal masses are very infrequent lesions that might occasionally present a diagnostic and therapeutic challenge to the doctor [7]. Three factors largely affect the chance of malignancy: the location of the mass, the patient’s age, and the existence or absence of symptoms. Most mediastinal tumors are benign, although those in the anterior compartment are more likely to be cancerous. Because most lymphomas and germ cell tumors (GCTs) manifest between the second and fourth decades of life, age is a significant indicator of malignancy. Finally, people who have symptoms are more likely to have cancer [4].

Excess hormones, cytokines, and antibodies are commonly responsible for these systemic symptoms. They took Posteroanterior and lateral chest radiographs as part of the first evaluation of a suspected mediastinal mass. This provides insight into the size of the mass, anatomical positioning, density, and composition. As well as identifying cystic, vascular, and soft-tissue structures, computed tomography (CT) scanning is used to better describe mediastinal masses and their relations to neighboring structures.

Magnetic Resonance Imaging (MRI) is used to rule out or evaluate the severity of a neurogenic tumor. MRI is useful for determining the degree of vascular invasion or cardiac involvement [8]. Even while nuclear scans and biochemical analyses can better define a lesion, a tissue diagnosis is almost always necessary. With this background, we did the current study to describe socio-demographic characteristics, symptoms, diagnosis, and location of mediastinal mass among the study participants.

## Materials and methods

Study design

This was a cross-sectional analytical study.

Study palace and study period

We conducted this study in a tertiary care center in Chennai over a three-year period from January 1, 2019 to December 31, 2022.

Study population

Patients admitted to a tertiary care hospital with an identified mediastinal mass diagnosed by CT scan during the study period.

Ethical clearance

We conducted this study after getting an ethical certificate from the institutional ethics committee (IEC number: CSP-MED/22/JAN/73/08).

Inclusion criteria

We included patients with an age above 16 years admitted to the tertiary care center in Chennai during the study period. The study included all patients with a mediastinal mass diagnosed by CT scan, with or without signs and symptoms of mediastinal compression.

Exclusion criteria

We excluded patients under the age of 16 and those whose information could not be retrieved from data sources.

Sampling technique and sample size

We included all the patients (convenient sampling) who met the eligibility criteria during the study period (three years) as study subjects. According to Aroor et al., only 3% of chest tumors were mediastinal tumors [1]. With this prevalence rate and 5% absolute error, the minimum sample needed for this study was 44 with a 95% confidence interval. We got the sample size by using the formula 3.84*p*q/d^2^, where p is prevalence, q is the complement of p, and d is an absolute error.

Data collection

We gathered all information pertaining to individuals diagnosed with mediastinal masses during the study period from hospital records. The case sheet contains demographic information about the patient. We also collected the case sheet for information about the patient’s chief complaint, medical history, and co-existing conditions. Blood parameters (complete blood count; CBC); liver function test (LFT); renal function test (RFT); fasting and postprandial blood sugar; and HbA1C; and pleural fluid parameters (total and differential count; sugar; protein; culture report) were all recorded in the laboratory register. The lab reports also included sputum AFB and GeneXpert data. The case document included the results of the x-ray and CT scan of the chest. From lab records, the histopathology report was taken.

Data analysis

We entered all data into Microsoft Excel (Microsoft Corporation, Redmond, WA), and then Statistical Package for Social Sciences (SPSS) 21 (IBM Corp., Armonk, NY) was used to perform statistical analysis. We used mean and standard deviation for all numerical data, while we used frequency and percentage for qualitative information. Chi-square analysis was used to determine whether there was a statistically significant association between the risk factors and the outcome variable (tumor type). If the P-value was less than 0.05, the results were significant.

## Results

We included about 44 study subjects in this study. Table 1 describes the demographic variables of the study participants. The mean age of the study participants was 41.11 years, with a high proportion of patients belonging to the age group of 21 to 30 years. Nearly 70% of the study participants were male.

**Table 1:** Socio-demographic characteristics of the study participants (n = 44)

S. No	Variables		Frequency	Percent
1	Age in years		Mean – 41.11 Standard deviation – 17.45	
2	Age group	Less than or equal to 20 years	4	9.1
		21 to 30 years	15	34.1
		31 to 40 years	6	13.6
		41 to 50 years	3	6.8
		51 to 60 years	10	22.7
		61 to 70 years	3	6.8
		Above 70 years	3	6.8
3	Gender	Female	13	29.5
		Male	31	70.5

Table 2 describes the symptoms of the study participants. Only about 54.5% of the study participants had symptoms because of a mediastinal mass. The most common local symptom felt by the patients was dyspnea, followed by a dry cough. The most common systemic symptom felt by the patients was weight loss, followed by fever. Most of the study participants (47.7%) had visited a healthcare center within one month of the onset of symptoms.

**Table 2:** Distribution of symptoms of mediastinal mass among the study participants (n = 44)

S. No	Variables		Frequency	Percent
1	Presence of symptoms	No	20	45.5
		Yes	24	54.5
2	Local symptoms	Chest pain	4	9
		Hemoptysis	2	4.5
		Cough with expectoration	5	11.3
		Dry cough	6	13.5
		Dyspnea	8	18.0
		Swelling in the neck	3	6.8
		No local symptoms	24	54.5
3	Systemic symptoms	Fever	8	18.0
		Weight loss	11	25.0
		Puffiness of face	3	6.8
		No systemic symptoms	26	59.1
4	Duration of symptom	Less than 1 month	21	47.7
		1 month to 1 year	20	45.5
		More than 1 year	3	6.8

Table 3 describes the radiological findings of the study participants. About 25% of the study participants had normal x-ray findings; others had mediastinal widening. About 4.5% of the patients had pleural effusion (predominantly transudate), as diagnosed by x-ray. Most of the study participants had mass in the anterior mediastinum, followed by the posterior mediastinum.

**Table 3:** Distribution of radiological findings of the study participants (n = 44)

S. No	Variables		Frequency	Percent
1	Chest X-ray finding	Mediastinal widening	33	75.0
		Normal finding	11	25.0
2	Presence of pleural effusion	No	42	95.5
		Yes	2	4.5
3	Site of mass	Anterior mediastinum	41	93.2
		Middle mediastinum	1	2.3
		Posterior mediastinum	2	4.5

Table 4 describes the broad classification of the pathological diagnoses of the study participants. Most (about 72.7%) of the study participants had malignant mediastinal masses.

**Table 4:** Description of broad classification of pathological diagnosis of the study participants (n = 44)

S. No	Pathological diagnosis	Frequency	Percent
1	Benign	12	27.3
2	Malignant	32	72.7

Table 5 describes about the pathological diagnosis of the study participants. among 44 cases, most frequently encountered pathological entity was non-caseating granulomatous inflammation, probably sarcoidosis. In this study, the most common benign diagnosis was non-granulomatous inflammation whereas the most common malignant diagnosis was B-cell non-Hodgkin's lymphoma. Of the total 44 individual, one patient had Ewing’s sarcoma, small cell carcinoma, spindle cell neoplasm, and synovial sarcoma each.

**Table 5:** Pathological diagnosis of the study participants (n = 44)

S. No	Pathological diagnosis	Frequency	Percent
1	Ewing’s sarcoma	1	2.3
2	Granulomatous inflammation - tuberculosis	3	6.8
3	HL mixed cellularity	2	4.5
4	Hodgkin’s lymphoma - sclerosis type	4	9.1
5	Mixed germ cell tumor	2	4.5
6	Myxoid liposarcoma	4	9.1
7	Non-granulomatous inflammation	2	4.5
8	Non-caseating granulomatous inflammation - sarcoid	7	15.9
9	Non-Hodgkin’s lymphoma - B-cell type	6	13.6
10	Small cell carcinoma	1	2.3
11	Schwannoma with vimentin	1	2.3
12	Spindle cell neoplasm	1	2.3
13	Squamous cell carcinoma	3	6.8
14	Synovial sarcoma	1	2.3
15	Thymoma	6	13.6

Table 6 describes the pathological diagnosis of the study participants according to the compartment where they were located. In the anterior mediastinum, the most common mass was non-caseating granulomatous inflammation, followed by non-Hodgkin’s lymphoma. The third common mass was thymoma. Only one patient had a middle mediastinal mass, which was diagnosed as a thymoma. Two patients had posterior mediastinal masses: one as small cell carcinoma and the other as spindle cell neoplasm.

**Table 6:** Pathological diagnosis of the study participants according to their site of mass (n = 44)

Site of mass	Pathological diagnosis	Frequency	Percent
Anterior mediastinum	Ewing’s sarcoma	1	2.4
	Granulomatous inflammation - tuberculosis	3	7.3
	HL mixed cellularity	2	4.9
	Hodgkin’s lymphoma - sclerosis type	4	9.8
	Mixed germ cell tumor	2	4.9
	Myxoid liposarcoma	4	9.8
	Non-granulomatous inflammation	2	4.9
	Non-caseating granulomatous inflammation - sarcoid	7	17.1
	Non-Hodgkin’s lymphoma - B-cell type	6	14.6
	Schwannoma with vimentin	1	2.4
	Squamous cell carcinoma	3	7.3
	Synovial sarcoma	1	2.4
	Thymoma	5	12.2
Middle mediastinum	Thymoma	1	100.0
Posterior mediastinum	Small cell carcinoma	1	50.0
	Spindle cell neoplasm	1	50.0

Table 7 shows the relationship between tumor type and risk factors among study participants who had a pathological report. For age, people between the ages of 31 and 50 have a higher risk of developing a malignant tumor than others. They had a statistically significant association (P = 0.001) by using the Chi-square test. Similarly, those patients who had no symptoms had a high risk of having a malignant tumor with a statistically significant association (P = 0.039) by using the Chi-square test. Other factors (gender, duration of symptoms, presence of pleural effusion, and location of mass) had no statistically significant association.

**Table 7:** Association between malignancy and risk factors among the study participants (n = 44) *Fisher exact test value

S. No	Variables			Broad pathological diagnosis		Chi-square value	P-value
				Benign	Malignant		
1	Age group	Less than or equal to 20 years	Count	4	0	17.207*	0.001
			%	100.0%	0.0%		
		21 to 30 years	Count	2	13		
			%	13.3%	86.7%		
		31 to 40 years	Count	0	6		
			%	0.0%	100.0%		
		41 to 50 years	Count	0	3		
			%	0.0%	100.0%		
		51 to 60 years	Count	2	8		
			%	20.0%	80.0%		
		61 to 70 years	Count	2	1		
			%	66.7%	33.3%		
		Above 70 years	Count	2	1		
			%	66.7%	33.3%		
2	Gender	Female	Count	2	11	1.315^*^	0.489
			%	15.4%	84.6%		
		Male	Count	10	21		
			%	32.3%	67.7%		
3	Presence of symptoms	No	Count	2	18	5.515	0.039
			%	10.0%	90.0%		
		Yes	Count	10	14		
			%	41.7%	58.3%		
4	Duration of symptoms	1 month to 1 year	Count	7	13	1.391^*^	0.503
			%	35.0%	65.0%		
		Less than 1 month	Count	5	16		
			%	23.8%	76.2%		
		More than 1 year	Count	0	3		
			%	0.0%	100.0%		
5	Presence of pleural effusion	No	Count	11	31	0.546^*^	0.476
			%	26.2%	73.8%		
		Yes	Count	1	1		
			%	50.0%	50.0%		
6	Site of mass	Anterior mediastinum	Count	12	29	0.901^*^	1 .000
			%	29.3%	70.7%		
		Middle mediastinum	Count	0	1		
			%	0.0%	100.0%		
		Posterior mediastinum	Count	0	2		
			%	0.0%	100.0%		

## Discussion

According to the current study, we diagnosed approximately 22.7% of study participants with lymphoid malignancy (which includes both Hodgkin and non-Hodgkin lymphomas). The second common diagnosis found in our study was non-granulomatous inflammation, which was 20.4%. In our study, all cases of non-Hodgkin’s lymphoma were of the B-cell type. The third most common diagnosis found by the current study was thymoma, which accounted for 13.6% of all diagnoses. In contrast to our findings, a study conducted in Sri Lanka in 2022 discovered that the most common mass was a thymic lesion (31%), followed by lymphoma (18.2%) [9]. Another study found that most of the study participants were male [9], which supported our study results that most of the patients (70.5%) were male. One more study by Bekele et al. found similar results that about 67.1% of their study participants were male [10]. Our study also found that the most common diagnosis in the anterior compartment was a non-caseating granulomatous lesion. This result was challenged by a study by Saha et al., in India, where the most common diagnosis was found to be thymoma in the anterior compartment [11]. We discovered only one case of a middle mediastinal mass in our study, and we diagnosed it to be a thymoma.

In our study, most of the masses were malignant. Similar to our study results is a study by Aroor et al., in 2014, in India, in which they found that most of the cases (68.5%) were malignant [1]. Another study done in Iran by Vaziri et al. found a similar result, that about 73% of the mediastinal masses were malignant [12]. In contrast to our findings, Adegboye et al., in Nigeria concluded that most mediastinal masses (70.8%) were benign [13]. Another study, conducted by Davis et al., recorded about 42% mediastinal masses were malignant [4]. Another study by Aggarwal et al., in India, supports our evidence that most of the mediastinal masses were malignant [14]. Thus, these differences might be because of the difference in the geographical distribution, yet this needs further exploration. In our study, we found most of the cases in the third decade of life. Aroor et al. found a similar finding in 2014, in which they found that most of the cases (25.71%) were in the third decade of age [1]. Our study also found that the mean age of the study participants was 41 years, which was supported by another study done in Sri Lanka in 2022 by Mathangasinghe et al., where the mean age of the samples was 43 years [9]. Another study, done in 2016, by Aggarwal et al., in India, found similar results. They did this study among 116 patients with mediastinal masses and found that most of the patients belonged to the age group of 21 to 30 years [14].

The current study found that most of the mediastinal mass (93.2%) was present in the anterior compartment. A similar finding was found by Mathangasinghe et al., in 2022, in a study done in Sri Lanka, they diagnosed 55% of the patients with an anterior mediastinal mass [9]. One more study by Bekele et al. found similar results: 61.6% of their study participants have an anterior mediastinal mass [10]. Another study done in India by Aggarwal et al. recorded most of the masses in the anterior compartment [14]. In our study, the common symptom found was dyspnea, which was supported by Bekele et al., in a study done in 2013 [10]. In our study, the second common symptom was a dry cough, but the same study [10] gave a different result, where the second common symptom was chest pain. Besides the above finding, 4.5% of the patients had pleural effusion as a complication, which was found by x-ray. This discussion needs further exploration. Besides the above finding, our study also found that patients in the middle age group (aged 31-50 years) had a statistically significant association (P = 0.001). Similarly, those patients who had no symptoms had a statistically significant association (P = 0.039) by using the Chi-square test. Yet, this needs further exploration.

Limitation of the study

We could retrieve only data from the past three years and used in the analysis due to resource constraints. Data with a longer study duration may yield better results. We did not include the reports in the study results because the CT scan findings differed for each patient. Other than pleural effusion, we recorded no other complications in our study. The history written in the case sheet and the radiologist who interprets the imaging were different for most of the patients, which may involve inter-observer bias. This can influence the study results. Despite the limitations mentioned above, the current study discovered useful associations that may encourage future research studies.

## Conclusions

The most common tumor found in our study was lymphoma in which non-Hodgkin's lymphoma is more common than Hodgkin's lymphoma. It was followed by a noncaseating granulomatous lesion and a thymoma. Anterior compartments are most commonly involved. We found only one patient with a middle mediastinal mass which was found to be a thymoma. We discovered the risk factors to be male gender and the third decade of life, with dyspnea being the most common symptom, followed by a dry cough. Our study found 4.5% of the patients had pleural effusion as a complication.
